# An extraordinary wave of SARS-CoV2, the youngest and the least serious

**DOI:** 10.3389/fpubh.2023.1238045

**Published:** 2023-10-30

**Authors:** Elena Justribó, Alberto Falcon, Maria José Ruiz-Vert, Juliana Porque, Silvia Gros, Oriol Yuguero

**Affiliations:** ^1^ERLab, Research on Emergencies. IRBLLEIDA, Lleida, Spain; ^2^Department of Medicine and Surgery, Faculty of Medicine, University of Lleida, Lleida, Spain

**Keywords:** SARS-CoV2, Spain, youth, migrant, social determinants

## Abstract

The second wave of SARS-CoV2 in our health region began at the end of June 2020 coinciding with the widespread decrease in cases in all areas of Catalonia. This resulted in the relaxation of measures that coincided with the end of the school year and the arrival of seasonal workers for the fruit-picking campaign. This led to a rapid increase in cases that culminated in perimeter confinement of the Segrià in July 2020, the only such measure imposed in the whole of Spain that month. This increase in cases was attributed to the influx of agricultural workers with a change of trend in the age of persons affected that had until then consisted mainly of older people. Simultaneously, in other areas of Catalonia the number of cases had dropped dramatically.

## Background

The year 2020 will be remembered by all humanity as the year of the SARS-CoV2 pandemic (1) with all that it has entailed for society beyond health-related aspects (2). In Catalonia, Spain, between 2020 and 2022, we can break the pandemic down into seven major waves, according to the occurrence of peaks in the number of cases. The data studied belong to the period spanning from 1 March 2020 to 30 November 2021, assuming the case bias of the first three months secondary to underdiagnosis due to the lack of diagnostic tests.

The total number of cases in Catalonia during this period was 883,766, with 361,549 corresponding to 2020 and 522,217 to 2021 out of a total population of around 7 million inhabitants (3). The total number of cases in the Lleida health region in 2020 was 26,114, or 7.2% of the figure for Catalonia, and 29,055 in 2021, accounting for 5.5% of the total cases in Catalonia.

The Lleida region, in Catalonia, is an agricultural region with a population of about 400,000 people, which increases considerably during the summer months for the harvesting of various fruit, due to the influx of people who are hired at their place of origin and come to Lleida to work. The massive increase in cases that was related to the arrival of seasonal workers for the fruit hest and processing in the large fruit cooperatives of the area led to this region’s perimeter closure and confinement. The confinement took place from 4–20 July 2020, in an area where almost every day the temperature exceeds 35 degrees. In a study published in 2021 (4), we analyzed the characteristics of immigrant patients who had suffered SARS-CoV2 during this wave.

The purpose of this review is to evaluate the characteristics of this extraordinary wave that had a social and economic impact on this region of Spain.

## Materials and methods

A retrospective, descriptive study of COVID cases between 1 March 2020 and 30 November 2021 in Catalonia and the Lleida Health Region. The Lleida health region has about 400,000 inhabitants. All patients were included and no exclusion criteria were applied.

### Data source

Data were taken from two different information systems. Firstly, patients registered with the public system who consulted their family doctor or an emergency department by means of the Electronic clinical workstation programs. Secondly, all cases of SARS-CoV-2 notified to our region’s public health agencies. Thus, we managed to detect almost all cases, even those displaying few symptoms or who self-diagnosed by using an antigen test.

### Variables

The variables analyzed were demographic, age and gender, and confirmed diagnosis of COVID. A descriptive analysis was carried out with mean frequency analyzes, with a 95% confidence interval (CI).

The study was approved by the Ethics Committee (CEIC) of the IRBLLEIDA.

## Results

In this second wave in Catalonia there were 121,273 confirmed cases of SARS-CoV2. Lleida health region recorded 9.2% of cases (11,201) and was the second health region insofar as cases, after metropolitan Barcelona, which recorded 70.19% (85,112). The list is rounded off by the Terres de l ‘Ebre region which only recorded 0.79% (965) during the same period. Table 1 sets out the data.

**Table 1:** Patients attended to by waves in Catalonia.

COVID-19	2020	2021	TOTAL	1st wave 2020	2nd wave 2020	3rd wave 2020	4th wave 2020–2021	5th wave 2021
No. cases in the Lleida region	26,114	29,055	55,169	2,592	11,201	19,749	11,650	9,567
No. cases in Catalonia	361,549	522,217	883,766	54,609	121,273	248,501	200,606	110,081
No. cases in Camp de Tarragona	23,745	36,111	59,856	1,731	5,088	17,615	14,687	6,144
No. cases in Terres de l’Ebre	5,765	8,530	14,295	163	965	4,734	4,471	1,168
No. cases in Girona	41,430	57,673	99,103	4,996	9,875	28,092	23,436	17,474
No. cases in Central Catalonia	27,484	37,765	65,249	5,606	8,070	16,558	15,528	11,047
No. cases in Barcelona	236,699	354,897	591,596	39,506	85,122	161,474	130,785	67,568
Unclassified	312	186	498	15	177	279	49	113

Of these 11,201 COVID cases, in this second wave, 1,618 patients required assistance from a hospital Emergency Service. However, in this wave, the Lleida health region made 623 admissions, the second lowest with respect to the all of the waves globally.

**Figure 1:**
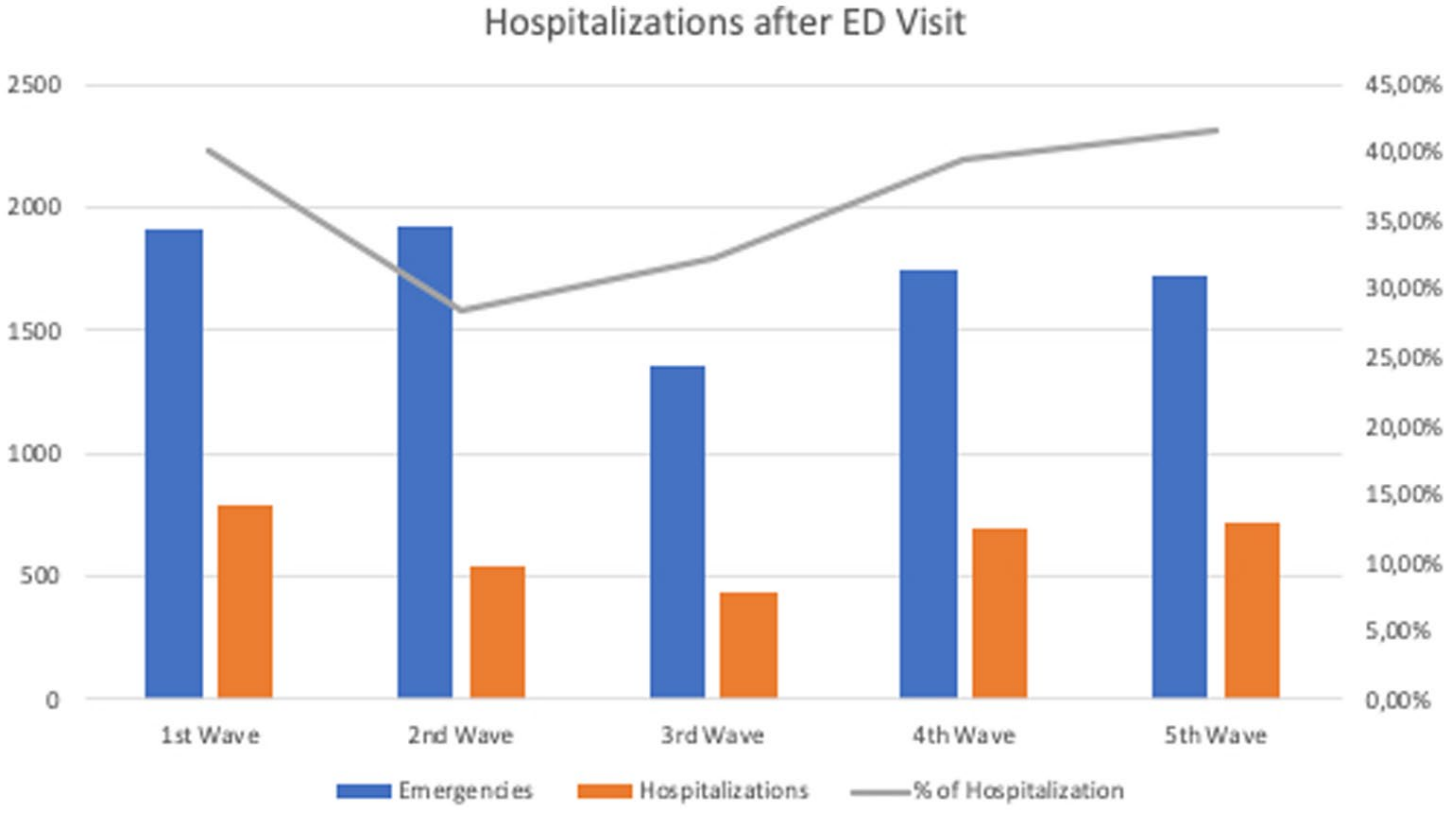
Hospitalizations of patients after Emergency Department visits during different waves.

Table 2 displays the emergencies for SARS-CoV2 attended to during the different waves. Can be seen that although we do not obtain statistical significance, the wave with the greatest influx of emergencies of young patients was the second wave. Figure 1 shows the number of emergencies and those requiring admission, which reveals how, compared to the first wave, patients required **fewer** hospitalization.

**Table 2:** Emergencies attended to by age and gender.

	<19 years		20–39		40–59		>60	
	Men	Women	Men	Women	Men	Women	Men	Women
First Wave	19	28	172	138	300	388	362	509
Second Wave	52	41	260	172	303	321	352	389
Third Wave	42	29	111	67	169	211	190	368
Fourth Wave	32	36	86	95	225	285	450	345
Fifth Wave	31	43	123	94	221	359	372	580

In this wave compared to the others, the two groups with an increase in cases are the group under 19 years of age and the group between 20 and 39 years of age. In the 20 to 39 years bracket, the increase is noted in both women and men and is far more **notable** in women than in men. In the under-19 age group, this increase is undergone by both sexes with a discreet increase in men.

## Discussion

The second wave of SARS-CoV2 in the Lleida Health Region led to an increase in cases among young people, especially women. Although the data for the second wave go up until September, the number of cases and emergency care and hospitalizations were concentrated in the first days of July, which brought about the perimeter closure of our region.

In the under-19 years age group, the distribution of cases is similar to the rest of the country. Probably due to the aforementioned causes: the relaxation of restrictions, the end of the academic course and graduation trips contributed to the increase in cases compared to the other waves. During the first months of the pandemic, schoolchildren were confined **with severe** measures and virtually no social interaction. It should also be added that people who come for the fruit-picking season usually belong to the next age bracket.

The main peculiarity is that in this second wave, the highest number of patients in the 20 to 39 age bracket occurred in the Lleida health region.

We believe it is important to mention the agricultural characteristics of this region. Lleida is one of the agricultural regions with the largest farms (5). On the one hand there are farms, where a greater effort is required. There are large extensions of fruit trees where the main activity is harvesting. The workers on these farms have low academic qualifications and in some cases poor economic conditions. On the other hand, there are cold stores where the fruit must be stored and prepared for transport and sale. This work in closed environments has better conditions. However, in many cases, long periods of time are spent in the same spaces where they live in the workplace, in the rest areas and dressing rooms. Even housing provided by the company itself. This will be important to understand some of the characteristics of this wave.

The arrival of seasonal workers in Lleida begins at the end of June and this has been the case year after year for many decades. This arrival is continuous with movements of people depending on the fruit variety to be harvested *in situ*, i.e., in orchards. This work is mainly carried out by men. The harvesting work requires transport to the fields that is usually by small vehicles, vans and also by bicycle, since many of these people usually live near the area, even in the local farmhouses. The work is performed basically outdoors and with little contact. It could be believed that there was a greater number of cases given that many of the men were in an irregular situation and did not want to come under the radar of the system for fear of losing their jobs. In fact, the COVID pandemic revealed some serious social situations in certain productive sectors of our country (6). For example, the poor housing conditions of most temporary workers. The same applies to other countries with flows of migrant workers (7).

We also find all the seasonal workers who come to work in the same period in the large cooperatives to pack and classify the harvested fruit. This work is mainly carried out by women. These large cooperatives are spread across many villages in the area and transport is mainly by bus, which involves close contact of many people over a longer period of exposure. This work is carried out indoors with many people in close contact, which could enhance the spread of the disease.

Our study has limitations. Especially when it comes to generalizing and comparing with related research on outbreaks in migrant/seasonal workers elsewhere in Spain. There are few articles on SARS-CoV2 infections in the seasonal immigrant population. Most of them talk about the conditions of migrants already established in Spain, not those who come for the summer campaigns. Furthermore, the observational design is not the best for this type of study, but we believe that the data obtained are remarkable.

This wave mostly affected young patients, and although the absolute numbers of cases was not higher than in metropolitan Barcelona, the incidence was very high in a period of just a few days. In addition, most patients had no risk factors, so the hospitalization rate was very low. Restrictive measures managed to slow down the number of cases, and when the number of cases of SARS-CoV2 began to increase in the rest of the country, in our region it began to decline. We can thus emphasize that this second wave was extraordinary insofar as patient characteristics are concerned as they were younger and required fewer admissions, a trait that was not repeated in any other wave.

## Data availability statement

The original contributions presented in the study are included in the article/supplementary materials, further inquiries can be directed to the corresponding author.

## Ethics statement

The studies involving humans were approved by CEIM Hospital Universitari Arnau de Vilanova de Lleida. The studies were conducted in accordance with the local legislation and institutional requirements. Written informed consent from the participants was not required due to the retrospective characteristics of the study.

## Author contributions

EJ, AF, and MV did the analysis and database. SG and JP wrote the main part of the manuscript. OY supervised the project and review the manuscript. All authors contributed to the article and approved the submitted version.
